# Variable Impedance Control for Force Tracking in Multi-Mode Robotic Back Massage

**DOI:** 10.3390/s26134115

**Published:** 2026-06-29

**Authors:** Jingbo Xu, Chong Ren, Xiangjie Kong, Silu Chen

**Affiliations:** 1 School of Medical Devices, Zhejiang Pharmaceutical University, Ningbo 315500, China; xujb@zjpc.net.cn (J.X.); renc14356@gmail.com (C.R.); 2 Ningbo Institute of Materials Technology and Engineering, Chinese Academy of Sciences, Ningbo 315201, China; chensilu@nimte.ac.cn

**Keywords:** variable impedance control, admittance control, force control, robotic massage, closed architecture, Traditional Chinese Medicine

## Abstract

Achieving safe physical interaction on the human back is challenging due to respiratory rhythms, complex topography, and varying tissue stiffness. To enable compliant force tracking within commercial closed position-control robot architectures, this paper presents an adaptive variable damping admittance control framework driven by multi-dimensional force sensor feedback. A stiffness-free admittance model is constructed to eliminate steady-state tracking errors, integrated with a nonlinear adaptive damping law that sensitively responds to real-time force sensor measurements. This mechanism rapidly dissipates dynamic impact energy during contacts while maintaining low impedance during steady state. Validated via a high-fidelity MATLAB R2024b-CoppeliaSim co-simulation platform replicating Traditional Chinese Medicine (TCM) manipulations, the proposed sensor-driven strategy significantly improves force tracking fidelity over traditional fixed-parameter control. Quantitative results demonstrate that across all complex therapeutic waveforms, the root mean square error (RMSE) remains below 0.42 N, the mean absolute error (MAE) is within 0.32 N, and the squared correlation coefficient ($r^{2}$) exceeds 0.97. These findings confirm the high efficiency and clinical potential of the proposed framework.

## 1. Introduction

With the global acceleration of population aging, the incidence of chronic musculoskeletal disorders has shown a significant upward trend, with low back pain (LBP) affecting approximately 7.5% of the global population [1,2]. The Traditional Chinese Medicine (TCM) *Tuina* (medical massage) plays a vital rehabilitative role by relaxing muscles, promoting circulation, and alleviating spasms [3]. However, training qualified Tuina practitioners requires long educational cycles, and clinical massage is a highly repetitive, labor-intensive physical activity that often causes occupational injuries to therapists [4]. This supply–demand imbalance severely restricts the standardization and availability of Tuina [5]. Consequently, utilizing medical rehabilitation robots with multi-dimensional sensing and adaptive force control to assist or replace human therapists has become a crucial engineering demand [6].

Tuina targets a complex, multi-layered “skin–joint–muscle–fascia” coupling system. Biomechanically, local pressure and shear forces stretch hypertonic paraspinal muscles (such as the erector spinae), breaking adhesions and restoring the spinal range of motion (ROM). Biophysically, compression deforms the blood and lymphatic vessels, improving local microcirculation to clear inflammatory cytokines (such as TNF-α and IL-6) and regulate extracellular matrix metabolism. Crucially, the thoracolumbar fascia (TLF), which stabilizes the spine and transmits force [7], exhibits altered mechanics in chronic low back pain (LBP), with thickness increasing by 25% and shear strain (gliding between layers) dropping by 20% [8]. Robotic massage restores TLF sliding, anatomically blocking pain signals. Furthermore, moderate-pressure massage stimulates the dermal mechanoreceptors, activating the parasympathetic nervous system (PNS) via vagal afferents [9]. This autonomic shift reduces heart rate, lowers cortisol levels, and elevates heart rate variability (HRV) [10]. Through the Gate Control Theory, these non-nociceptive mechanical signals block pain in the spinal dorsal horn while releasing endorphins and dopamine. Randomized controlled trials (RCTs) statistically prove that standardized Tuina reduces Visual Analog Scale (VAS) scores by 2.5–3.1 points and Oswestry Disability Index (ODI) scores by 10–15 points, achieving high patient satisfaction (up to 91.9%) [11]. This underscores the clinical value of precise force control to avoid rigid impact injuries [12].

Deploying Tuina on a patient’s back is challenging because the human back is an unstructured, variable viscoelastic environment with geometric complexities and breathing-induced displacement [13]. Pure position-feedback control risks generating destructive impact forces when sliding over bony landmarks (such as the scapula), causing secondary injury [14]. However, most commercial collaborative robots possess closed control architectures that encapsulate low-level joint torque loops and only expose Cartesian position command interfaces [15], treating external forces as disturbances to suppress [16]. Under this hardware constraint, admittance control based on an impedance reference model is the optimal path for bypass, calculating Cartesian position corrections from external force input [17]. Yet, conventional constant-parameter admittance control is restricted by the trade-off between transparency and stability: low damping parameters improve force tracking speed but trigger high-frequency limit-cycle oscillations on stiff bony regions, whereas high damping guarantees contact stability but introduces severe phase lag, failing to track respiratory movements [18]. Thus, designing a variable impedance strategy that guarantees stability during rigid collisions while maintaining tracking bandwidth on closed architectures remains a major challenge.

To address the aforementioned technical bottlenecks, this paper is dedicated to proposing a variable impedance admittance control algorithm that breaks through the limitations of closed motion control architectures, and applying it to multi-mode TCM rehabilitation massage scenarios. The main contributions of this paper can be summarized as follows:

1. An adaptive variable damping admittance control algorithm is proposed for robotic arms with closed position servo characteristics. Starting from the physical essence of energy evolution, this algorithm circumvents the reliance of traditional hybrid control on underlying torque interfaces, achieving precise force control solely by modifying the target trajectory in Cartesian space.
2. To eliminate the steady-state force error, the virtual stiffness term is discarded in the variable impedance control algorithm. Instead, the implicit Euler method is employed to reconstruct the dynamics, preventing numerical divergence under rigid contact [18]. Concurrently, an adaptive damping control law is designed based on force dynamics, ensuring stability upon collision and expanding the force control bandwidth.
3. A stepped synchronous high-fidelity co-simulation platform is set up based on MATLAB and CoppeliaSim. The mappings from traditional typical modes of TCM massage into force control tasks are set up with time-varying trajectories, such as fixed points, sine waves, triangle waves, trapezoidal waves, and square waves [19,20].

Systematic simulations conducted on planar, inclined, and complex 3D bionic curved surfaces comprehensively verify the effectiveness and outstanding robustness of the proposed variable impedance control algorithm in complex unstructured environments.

## 2. Related Works

### 2.1. Compliant Physical Interaction

Robotic interaction with physical environments can be broadly categorized into passive compliance and active compliance. Passive compliance relies on physical hardware, such as springs and dampers, which react rapidly but lack the programmable adaptability required for precision medical interventions [21]. Active compliance regulates motor behavior through control algorithms to shape the robot’s external characteristics [22]. The most prominent paradigm is impedance control, which specifies a dynamic target relationship, typically modeled as a virtual mass–spring–damper system between the external force and the tracking error [23].

Impedance control is generally realized in two ways: torque-based direct impedance control [24] and position-based admittance control [25]. Torque-based impedance control directly commands joint torques based on joint-space force measurements [26]. While it provides natural back-drivability and high transparency, it heavily relies on highly accurate robot dynamic models (accounting for gravity, friction, and joint inertia) and is sensitive to nonlinear gear friction [27]. Position-based admittance control is highly suitable for commercial collaborative and industrial robots that do not expose low-level torque interfaces [23]. It reads external forces and computes a commanded position correction, utilizing the high-precision inner position loop of industrial manipulators. Admittance control also exhibits superior numerical robustness when the robot transitions from free space to constrained contact. Nevertheless, traditional admittance controllers utilize constant mass, spring, and damper coefficients [28]. When interacting with the back’s mixed rigid–soft topography (e.g., sliding from soft waist tissue to rigid scapulae), fixed-parameter controllers struggle to bridge the gap between transparency and contact stability.

### 2.2. Adaptive Variable Impedance/Admittance Control

To overcome fixed-parameter limits, variable impedance control (VIC) and variable admittance control (VAC) adjust reference parameters online [29]. Model-based VIC approaches rely on online estimation of environment stiffness and position via recursive least squares or observers to compute target parameters [30]. However, on highly nonlinear viscoelastic human tissue, localized linear models fail, and parameter estimation lag during fast respiratory or contact transitions can cause controller saturation or instability [31]. Conversely, model-free adaptive schemes adjust parameters directly using tracking errors [32]. An adaptive variable impedance control (AVIC) law is proposed to adjust stiffness and damping online using force errors to track dynamic force profiles on uncertain workpiece surfaces [33]. Although model-free methods bypass parameter estimation, they often require torque-level control access and can introduce high-frequency discretization noise under non-linear laws [18], limiting their direct application on closed position servo industrial manipulators [34].

### 2.3. Learning-Based Variable Impedance Control

Reinforcement learning (RL) enables robots to optimize interaction impedance through trial-and-error exploration without prior dynamic models [35,36]. Notably, an online reinforcement learning method to improve control adaptability in robot-aided rehabilitation (EAAI) is proposed with a Q-learning-based online RL framework for upper-limb rehabilitation [37]. This system adjusts radial stiffness and execution time based on patient kinematic and physiological feedback, enhancing movement smoothness and tracking accuracy. Continuous deep reinforcement learning (DRL) policies (e.g., DDPG, TD3) have also been explored to map force readings directly to continuous impedance matrices [38]. However, implementing RL-based variable impedance control in safety-critical back massage is hindered by three fatal flaws. The first is sample inefficiency and the sim-to-real gap. Notably, DRL policies require extensive exploration steps. Despite sim-to-real transfers, unpredictable breathing and individual anatomical variations necessitate online fine-tuning, introducing dangerous exploratory behaviors in direct human contact [38]. The second is the absence of safety guarantees. Specifically, stochastic RL exploration lacks passive physical constraints, risking negative stiffness/damping outputs that can cause severe vibrations and injure a patient’s ribs or spine [39]. The third is computational latency. Deep network inference overhead restricts control loops to 10–30 Hz, which is too slow to suppress millisecond-level transient shock loads during rigid bone collisions [40].

### 2.4. Massage Robots and Skin Contact Force Control

Because massage occurs directly on sensitive, non-homogeneous skin and muscle surfaces, it demands continuous, gentle force regulation and rapid adaptation to surface contours [8,41]. Various specialized platforms and force-control algorithms have been designed to meet these requirements [42,43]. At the structural level, a multi-fingered robot hand using hybrid impedance control is proposed, employing position-based impedance for lateral motions and force-based impedance for vertical compression [44]. However, strong dynamic coupling between orthogonal directions can cause the massage head to slip when traversing bony structures like the scapula. To maintain stable contact on flexible skin, GMM/GMR algorithms are utilized to fuse offline reinforcement learning strategies with online physical models, enhancing the algorithm’s robustness [45]. Additionally, autonomous back massage systems integrating 3D reconstruction and deep learning have progressed significantly. These systems utilize RGB-D cameras to reconstruct paraspinal point clouds, adjusting the normal orientation of the massage head while implementing constant force tracking via fixed-parameter impedance control. However, when sliding from soft waist tissue to rigid paraspinal bony structures, fixed parameters produce contact force overshoots of up to 4.96 N due to localized tissue stiffness variations [46]. Thus, a lightweight, non-heuristic, and provably passive variable impedance control scheme is highly desired [47].

## 3. Problem Formulation

In the system design and control law synthesis of the massage robot, a rigorous definition of the control objective and dynamic characteristics of the interactive environment forms the foundation for ensuring the algorithm’s analytical resolvability and engineering feasibility.

### 3.1. General Control Strategy and Objectives

Consider a general multi-axis robotic manipulator performing physical human–robot interaction (pHRI) in an unstructured environment. Let $x\in R^{m}$ represent the actual Cartesian pose (including 3D position and orientation) of the robot’s end-effector in the base coordinate frame, which can be derived from joint-space coordinates via forward kinematics:

(1) x=f(q)

where $q\in R^{n}$ denotes the joint angle vector, and n≥m. Its differential kinematics are described as $\dot{x}=J\left(q\right)\dot{q}$, where $J\left(q\right)\in R^{m\times n}$ is the Jacobian matrix.

In practical engineering applications, a vast majority of commercial robotic platforms strictly encapsulate their underlying joint torque loops and current loops, exposing only a high-gain position or velocity servo interface to secondary developers [17]. Under such constraints, the high-level higher-frequency inner loop acts as an ideal position integrator. Assuming the inner-loop bandwidth is sufficiently higher than the outer-loop force interaction frequency, the actual achieved Cartesian pose $x_{a}\left(t\right)$ can accurately track the desired command pose $x_{c}\left(t\right)$ without delay, i.e., $x_{a}\left(t\right)\approx x_{c}\left(t\right)$.

Consequently, the physical force-tracking control problem is equivalently transformed into a real-time trajectory planning problem in the Cartesian space. To achieve this, a position-based adaptive admittance control law, denoted as Φ, is introduced to govern the mechanical compliance characteristics along the contact’s normal direction.

Let $F_{d}\left(t\right)$ be the target therapeutic interaction force profile, and $F_{ext}\left(t\right)$ be the actual contact force measured by a force sensor. The general force tracking error is defined as

(2) $$e_{f}\left(t\right)=F_{d}\left(t\right)-F_{ext}\left(t\right)$$

The primary objective of the proposed control strategy is to design the admittance mapping Φ that takes the external force error $e_{f}\left(t\right)$ and its derivative ${\dot{e}}_{f}\left(t\right)$ as inputs, dynamically computing a normal position correction value $z_{c}\left(t\right)$ in real time, as follows:

(3) $$z_{c}\left(t\right)=\Phi \left(e_{f}\left(t\right),{\dot{e}}_{f}\left(t\right)\right)$$

This normal regulation command is then superimposed onto a pre-planned spatial task reference trajectory $x_{r}\left(t\right)$ (which handles tangential exploration and postural alignment) via a force-position hybrid decoupling matrix T:

(4) $$x_{c}\left(t\right)=x_{r}\left(t\right)+Tz_{c}\left(t\right)$$

The matrix T ensures that the normal force-regulation component $z_{c}\left(t\right)$ is projected into Cartesian space without interfering with the tangential position-tracking performance of $x_{r}\left(t\right)$.

To guarantee clinical safety and therapeutic efficacy, the control law Φ must satisfy the following three core performance objectives for any generic closed position-controlled robot:

1. **Asymptotic Force Convergence:** The steady-state force error must satisfy $\lim_{t\to \infty }∥e_{f}\left(t\right)∥$≤ϵ, converging into an allowable minimal neighborhood even under unknown environmental profiles.
2. **Contact Passivity and Stability:** The system must strictly satisfy Lyapunov stability conditions, effectively suppressing high-frequency oscillations or limit-cycle phenomena when contacting rigid unmodeled boundaries.
3. **Transient Energy Dissipation:** For target force profiles with sharp step transitions or high gradients, the system must guarantee smooth tracking with bounded transient overshoots.

Based on this general theoretical control framework, the specific experimental evaluation scenario, including the chosen robotic system and environmental properties, will be detailed in the following subsections.

### 3.2. Model of Human Back

The massage robot is interacting with the human back, which is a typical unstructured, complex, and unknown biomechanical system. During massage tasks, the soft tissues of the human back (skin, fascia, muscles) exhibit significant nonlinear viscoelastic characteristics [48]. Although high-order nonlinear contact models like the Hunt–Crossley model are more accurate in describing large-range deformations [49], they are not suitable for the synthesis and real-time online calculation of local controllers. Thus, a locally linearized Kelvin–Voigt model is used in the vicinity of the contact point to approximate the environment’s normal impedance characteristics.

Defining the actual contact force along the normal direction of the massage surface as $F_{ext}\left(t\right)$, it can be expressed as

(5) $$F_{ext}\left(t\right)=k_{h}\left(x_{a}\left(t\right)-x_{h}\left(t\right)\right)+B_{h}{\dot{x}}_{a}\left(t\right),$$

where $x_{a}\left(t\right)$ is the actual penetration position of the massage head in the normal direction; $k_{h}$ is the equivalent environment stiffness coefficient of the human back; $B_{h}$ is the equivalent environment damping coefficient; and $x_{h}\left(t\right)$ is the natural resting position of the skin surface, which is the equilibrium point without deformation.

In actual massage operations, it is impossible and unnecessary to know the impedance characteristics ($k_{h},B_{h}$) and morphological characteristics ($x_{h}\left(t\right)$) of the human back accurately. Specifically, variations between patients, different acupoints, and even the depth of the massage force can cause sudden cross-magnitude changes in $k_{h}$ (e.g., sliding from a soft fat layer to a hard scapula region) [50]. Meanwhile, the skin’s resting position $x_{h}\left(t\right)$ is also an unknown disturbance variable continuously changing over time due to chest undulations caused by the patient’s respiratory rhythm.

Therefore, the prerequisite for designing the force control algorithm is to have an adaptive and stable regulated system relying solely on real-time multi-dimensional force sensor feedback in the absence of accurate models for environmental stiffness and geometric morphology.

## 4. Variable Impedance Control for Force Tracking

**Figure 1:**
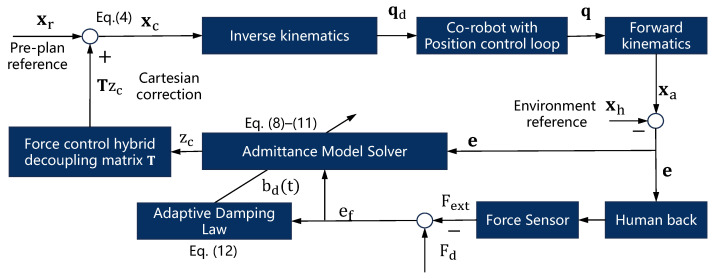
The control architecture for a massage robot with adaptive damping.

Having clarified the constraints of the closed control architecture and the time-varying characteristics of unknown environments, this chapter will derive the variable impedance (admittance) control algorithm used for force tracking, as shown in Figure 1, and provide a rigorous mathematical proof of its closed-loop stability.

### 4.1. Controller Design

To endow a robotic arm possessing only position-control interfaces with a compliant interaction capability, a virtual admittance model is introduced in the contact normal direction. In this paper, the positive direction of the normal position correction $z_{c}\left(t\right)$ is defined as the inward direction toward the human back. Therefore, increasing $z_{c}\left(t\right)$ increases the indentation depth of the massage end-effector and consequently increases the measured normal contact force. Since the force tracking error is defined as $e_{f}\left(t\right)=F_{d}\left(t\right)-F_{\mathrm{ext}}\left(t\right)$, a positive $e_{f}\left(t\right)$ indicates that the measured contact force is smaller than the desired force and the end-effector should move further along the pressing direction.

The second-order admittance model in the normal direction can be written as

(6) $$m_{d}{\ddot{z}}_{c}\left(t\right)+b_{d}{\dot{z}}_{c}\left(t\right)+k_{d}z_{c}\left(t\right)=e_{f}\left(t\right),$$

where $m_{d}$, $b_{d}$, and $k_{d}$ denote the virtual mass, virtual damping, and virtual stiffness in the contact’s normal direction, respectively, and $z_{c}\left(t\right)$ is the normal position correction generated by the outer-loop admittance model.

**Elimination of Steady-State Error with Stiffness-Free Model:** In rehabilitation massage tasks, the objective is to accurately regulate the absolute contact force rather than to impose a fixed virtual deformation. If the virtual stiffness term is retained, i.e., $k_{d}>0$, and the acceleration and velocity terms decay to zero, (6) reduces to

(7) $$\lim_{t\to \infty }e_{f}\left(t\right)=k_{d}z_{c}\left(t\right).$$

This relation indicates that a non-zero normal position correction generally leads to a non-zero steady-state force error. Eliminating this residual error would require accurate knowledge of the environment equilibrium position or an additional feedforward compensation term, which is difficult to obtain on the undulating and spatially varying human back. Therefore, the virtual stiffness term is removed by setting $k_{d}=0$, and the admittance model is reduced to a stiffness-free second-order mass–damper form.

For scalar control along the contact’s normal direction, the positive direction of the normal position correction $z_{c}\left(t\right)$ is defined as the inward direction on the human back. Therefore, increasing $z_{c}\left(t\right)$ increases the normal indentation of the massage end-effector and consequently increases the measured normal contact force. Consistent with the force tracking error defined in (2), i.e., $e_{f}\left(t\right)=F_{d}\left(t\right)-F_{\mathrm{ext}}\left(t\right)$, $e_{f}\left(t\right)>0$ indicates that the measured force is smaller than the desired force, and the controller should drive the end-effector further toward the human back. Conversely, $e_{f}\left(t\right)<0$ indicates excessive contact force, and the controller should reduce the indentation depth.

After removing the virtual stiffness term, the admittance dynamics in the normal direction are simplified as

(8) $$m_{d}{\ddot{z}}_{c}\left(t\right)+b_{d}\left(t\right){\dot{z}}_{c}\left(t\right)=e_{f}\left(t\right),$$

where $m_{d}>0$ denotes the virtual mass and $b_{d}\left(t\right)>0$ denotes the adaptive damping coefficient in the normal direction. This stiffness-free formulation introduces an integral effect from the force error to the position correction, thereby avoiding the non-zero steady-state force error that would be induced by a virtual stiffness term in absolute force tracking tasks.

In a digital controller, (8) is discretized by the implicit Euler method to improve numerical robustness during high-stiffness contact. At the *i*-th control cycle, the acceleration term is approximated using the current velocity ${\dot{z}}_{c,i}$ as

(9) $$m_{d}\frac{{\dot{z}}_{c,i}-{\dot{z}}_{c,i-1}}{\Delta t}+b_{d,i}{\dot{z}}_{c,i}=e_{f,i},$$

where Δt is the sampling period, $e_{f,i}=F_{d,i}-F_{\mathrm{ext},i}$, and $b_{d,i}$ is the damping coefficient updated at the current control cycle. Rearranging (9) yields the velocity update law

(10) $${\dot{z}}_{c,i}=\frac{m_{d}{\dot{z}}_{c,i-1}+e_{f,i}\Delta t}{m_{d}+b_{d,i}\Delta t}.$$

The normal position correction is then updated as

(11) $$z_{c,i}=z_{c,i-1}+{\dot{z}}_{c,i}\Delta t.$$

The physical meaning of (10) and (11) is consistent with the force regulation objective. When $F_{\mathrm{ext},i}<F_{d,i}$, the force error $e_{f,i}$ is positive and the updated velocity tends to increase $z_{c,i}$, thereby increasing the indentation depth and the contact force. When $F_{\mathrm{ext},i}>F_{d,i}$, the force error becomes negative and the admittance model drives $z_{c,i}$ in the opposite direction, reducing the indentation depth and relieving the excessive contact force. Moreover, the denominator $m_{d}+b_{d,i}\Delta t$ provides an implicit numerical damping effect, which improves robustness against numerical oscillation during contact with high-stiffness regions of the human back.

**Design of Adaptive Variable Damping Control Law:** For a force control system based on a stiffness-free model, its dynamic response quality depends entirely on the damping parameter $b_{d}\left(t\right)$. A single fixed damping parameter faces irreconcilable contradictions: weaker damping leads to high-frequency oscillation during contact transients, while greater damping causes excessive energy dissipation, making the system’s response to commands and environmental changes extremely sluggish. Therefore, the following adaptive variable damping control law directly driven by the force tracking error and its gradient is proposed:

(12) $$b_{d}\left(t\right)=b_{base}+\alpha \left|e_{f}\left(t\right)\right|+\beta \max\left(0,e_{f}\left(t\right)\;\cdot \;{\dot{e}}_{f}\left(t\right)\right)$$

where $b_{base}$ is the minimum baseline damping to guarantee fundamental system stability, t=iΔt, and α and β are positive adjustment gains. The physical mechanism of this algorithm is as follows:

1. **Steady-State Low Impedance:** When the actual force approaches the desired force ($e_{f}\approx 0$), the system is primarily dominated by $b_{base}$, maintaining a low-damping state to ensure the sensitivity of the massage process, thereby rapidly responding to interference from back surface curvatures and respiratory undulations [1,30].
2. **High Collision Dissipation:** When the robotic arm makes contact with an area with a sudden stiffness change (causing severe overshoot), the force error surges and continues to worsen (i.e., $e_{f}$ and ${\dot{e}}_{f}$ have the same sign), activating the βmax(·) term, which causes the virtual damping to spike instantaneously. The system instantly transforms into a high-dissipation energy field, forcibly absorbing and smoothing out the inertial momentum generated by rigid collisions.
3. **Unimpeded Convergence:** When the force error is large but converging towards zero ($e_{f}\;\cdot \;{\dot{e}}_{f}<0$), the penalty term becomes inactive, and the system moderately reduces damping to avoid excessive lag, accelerating seamless modal transitions.

### 4.2. Stability Analysis

To establish the closed-loop stability of the proposed adaptive variable damping admittance controller, the local normal contact dynamics are considered. The positive direction of the normal position correction $z_{c}\left(t\right)$ is defined as the inward direction toward the human back, such that an increase in $z_{c}\left(t\right)$ increases the normal contact force. Accordingly, the force tracking error is defined as

(13) $$e_{f}\left(t\right)=F_{d}\left(t\right)-F_{\mathrm{ext}}\left(t\right).$$

This sign convention is consistent with the physical regulation objective, e.g., when the measured contact force is smaller than the desired force, $e_{f}\left(t\right)>0$ drives the admittance model to increase the normal indentation. Conversely, when the measured force is larger than the desired force, $e_{f}\left(t\right)<0$ drives the end-effector to retract.

The following assumptions are made for the stability analysis.

**Assumption** **1.** *The robot inner position-control loop has a sufficiently high bandwidth, such that the actual Cartesian command can be accurately tracked, i.e., $x_{a}\left(t\right)\approx x_{c}\left(t\right)$.*

**Assumption** **2.** *Around a local contact point, the human back can be approximated by a passive linear elastic environment in the normal direction as follows:*

(14) $$F_{\mathrm{ext}}\left(t\right)=k_{h}z_{c}\left(t\right),$$

*where $k_{h}$ is unknown but positive and bounded, i.e., $0<k_{\min}\leq k_{h}\leq k_{\max}$.*

**Assumption** **3.** *During the local regulation interval considered in the stability proof, the desired force is constant or piecewise constant.*

Under Assumption 2, the force tracking error can be written as

(15) $$e_{f}\left(t\right)=F_{d}-k_{h}z_{c}\left(t\right).$$

Since $F_{d}$ is constant within the considered regulation interval, one has

(16) $${\dot{e}}_{f}\left(t\right)=-k_{h}{\dot{z}}_{c}\left(t\right),\;{\ddot{e}}_{f}\left(t\right)=-k_{h}{\ddot{z}}_{c}\left(t\right).$$

The proposed stiffness-free admittance model in the normal direction is given by

(17) $$m_{d}{\ddot{z}}_{c}\left(t\right)+b_{d}\left(t\right){\dot{z}}_{c}\left(t\right)=e_{f}\left(t\right),$$

where $m_{d}>0$ is the virtual mass and $b_{d}\left(t\right)>0$ is the adaptive damping coefficient. Substituting (16) into (17) yields the following nonlinear closed-loop force-error dynamics as

(18) $$\frac{m_{d}}{k_{h}}{\ddot{e}}_{f}\left(t\right)+\frac{b_{d}\left(t\right)}{k_{h}}{\dot{e}}_{f}\left(t\right)+e_{f}\left(t\right)=0.$$

This has the form of a damped second-order system with positive inertia, damping, and stiffness-like terms, suitable for Lyapunov-based stability analysis. Thus, the following theorem holds.

**Theorem** **1.** *Consider the nonlinear force error dynamics *(18)* if the adaptive damping law is designed as*

(19) $$b_{d}\left(t\right)=b_{\mathrm{base}}+\alpha \left|e_{f}\left(t\right)\right|+\beta \max\left(0,e_{f}\left(t\right){\dot{e}}_{f}\left(t\right)\right),$$

*where $b_{\mathrm{base}}>0$, α≥0, and β≥0. Consequently, the force tracking error asymptotically converges to zero as follows:*

(20) $$\lim_{t\to \infty }e_{f}\left(t\right)=0,\;\lim_{t\to \infty }{\dot{e}}_{f}\left(t\right)=0.$$

**Proof.**  Consider the following Lyapunov function candidate:

(21) $$V\left(e_{f},{\dot{e}}_{f}\right)=\frac{1}{2}\frac{m_{d}}{k_{h}^{2}}{\dot{e}}_{f}^{2}+\frac{1}{2k_{e}}e_{f}^{2}.$$

Because $m_{d}>0$ and $k_{h}>0$, $V(e_{f},{\dot{e}}_{f})$ is positive, definite, and radially unbounded with respect to $\left(e_{f},{\dot{e}}_{f}\right)$. Taking the time derivative of (21) gives

(22) $$\dot{V}=\frac{m_{d}}{k_{h}^{2}}{\dot{e}}_{f}{\ddot{e}}_{f}+\frac{1}{k_{h}}e_{f}{\dot{e}}_{f}.$$

From (18), one obtains

(23) $$\frac{m_{d}}{k_{h}^{2}}{\ddot{e}}_{f}=-\frac{b_{d}\left(t\right)}{k_{h}^{2}}{\dot{e}}_{f}-\frac{1}{k_{h}}e_{f}.$$

Substituting (23) into (22) leads to

(24) $$\dot{V}={\dot{e}}_{f}\left(-\frac{b_{d}\left(t\right)}{k_{h}^{2}}{\dot{e}}_{f}-\frac{1}{k_{h}}e_{f}\right)+\frac{1}{k_{h}}e_{f}{\dot{e}}_{f}=-\frac{b_{d}\left(t\right)}{k_{h}^{2}}{\dot{e}}_{f}^{2}.$$

Using (28), it follows that

(25) $$\dot{V}\leq -\frac{b_{\mathrm{base}}}{k_{h}^{2}}{\dot{e}}_{f}^{2}\leq 0.$$

Therefore, $e_{f}\left(t\right)$ and ${\dot{e}}_{f}\left(t\right)$ are bounded, and the closed-loop system is stable in the sense of Lyapunov.

To further prove asymptotic convergence, LaSalle’s invariance principle is applied. The set where $\dot{V}=0$ is

(26) $$S=\{\left(e_{f},{\dot{e}}_{f}\right)|{\dot{e}}_{f}=0\}.$$

For the trajectory to remain invariant in S, it must also satisfy ${\ddot{e}}_{f}=0$. Substituting ${\dot{e}}_{f}=0$ and ${\ddot{e}}_{f}=0$ into (18) gives

(27) $$e_{f}=0.$$

Thus, the largest invariant set contained in S is the origin $\left(e_{f},{\dot{e}}_{f}\right)=\left(0,0\right)$. Consequently, (20) holds. This proves that the proposed adaptive variable damping admittance controller guarantees asymptotic force regulation under unknown but positive bounded environment stiffness. This proves Theorem 1. In addition, from (19),

(28) $$b_{d}\left(t\right)\geq b_{\mathrm{base}}>0$$

holds all the time. □

**Remark on time-varying desired forces:** The above proof establishes asymptotic convergence for constant or piecewise-constant desired force segments. For time-varying desired forces, such as sinusoidal, triangular, or trapezoidal force profiles, the error dynamics become

(29) $$\frac{m_{d}}{k_{h}}{\ddot{e}}_{f}\left(t\right)+\frac{b_{d}\left(t\right)}{k_{h}}{\dot{e}}_{f}\left(t\right)+e_{f}\left(t\right)=\frac{m_{d}}{k_{h}}{\ddot{F}}_{d}\left(t\right)+\frac{b_{d}\left(t\right)}{k_{h}}{\dot{F}}_{d}\left(t\right).$$

Therefore, the closed-loop system can be interpreted as a stable second-order force-error system driven by the bounded derivatives of the desired force. If ${\dot{F}}_{d}\left(t\right)$ and ${\ddot{F}}_{d}\left(t\right)$ are bounded, then $e_{f}\left(t\right)$ and ${\dot{e}}_{f}\left(t\right)$ remain bounded. The tracking accuracy for these time-varying massage profiles is quantitatively evaluated in the simulation section using RMSE, MAE, maximum absolute error, and the squared correlation coefficient.

## 5. Validation by Simulation

Theoretical derivations must withstand the test of physical dynamic constraints. To verify the smoothness of the aforementioned dual-quaternion kinematic planning and the robustness boundaries of the adaptive variable damping strategy in unstructured environments, this section utilizes an advanced virtual physics engine to conduct rigorous co-simulation experiments.

### 5.1. Setup of Simulation Model

This study realized mathematical simulations in a single software and constructed a high-fidelity co-simulation architecture based on MATLAB and CoppeliaSim to replicate true physical interaction responses.

**Figure 2:**
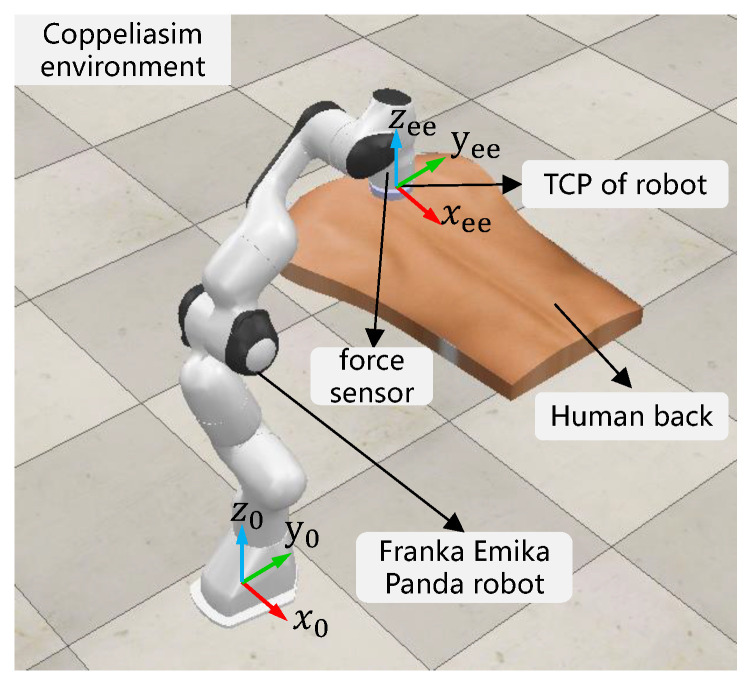
The 7-DOF co-robot being used for this study.

- **Simulation Environment and Robot Model:** A 3D dynamic model of the Franka Emika Panda collaborative robotic arm, as shown in Figure 2 and its complete URDF parameters were introduced into the CoppeliaSim environment. To align with the closed control architecture constraints from theoretical derivation, the engine’s underlying torque feedforward control interfaces were disabled. Instead, an Inverse Kinematics solver based on Damped Least Squares (DLS) was strictly called to generate joint position control sequences, effectively applying a barrier reflective of real industrial environments to the algorithm.
- **Design of Force-Sensing Interface:** A hemispherical massage head with a built-in 6D force/torque virtual sensor module was mounted on the robotic arm’s end flange. To ensure the accuracy of force calculation, an attitude transformation matrix mapping was constructed in real time between the sensor and world coordinate systems, and Gaussian white noise was superimposed to simulate the high-frequency interference typical of hardware sensors.
- **Model of Interaction Environment:** The back, for massage tasks, is not flat and uniform. In the simulation scene, physical rigid bodies possessing true-contact mechanical properties (friction coefficients, stiffness, restitution coefficients of damping) were utilized to construct three distinct biomechanical simulated environments:
  – An orthogonal plane;
  – A time-varying inclined plane;
  – Complex curved surfaces with highly unstructured features.
  The last one simulates abrupt curvature changes of the scapula and spine on the human back.
- **Synchronous Communication by ZeroMQ in Microseconds:** At the data link layer, a full-stack Stepped Synchronization architecture based on ZeroMQ was introduced. The discrete control law calculations in MATLAB and dynamic rendering in CoppeliaSim were forcefully clock-aligned, completely eliminating network jitter caused by asynchronous communication. This provided physics engine-level assurance for the numerical stability of the Implicit Euler method at millisecond-level cycles.

### 5.2. Design of Simulation

To verify the force tracking capability of the proposed adaptive variable damping admittance controller under massage-oriented contact conditions, a high-fidelity MATLAB–CoppeliaSim co-simulation was designed. Different from a single constant-force benchmark, the simulation was constructed to simultaneously examine three coupled difficulties that commonly appear in robotic back massage, i.e., time-varying desired force, tangential massage motion over a curved back surface, and spatially varying human back stiffness. Therefore, the validation was organized from both the task side and the environment side.

**Figure 3:**
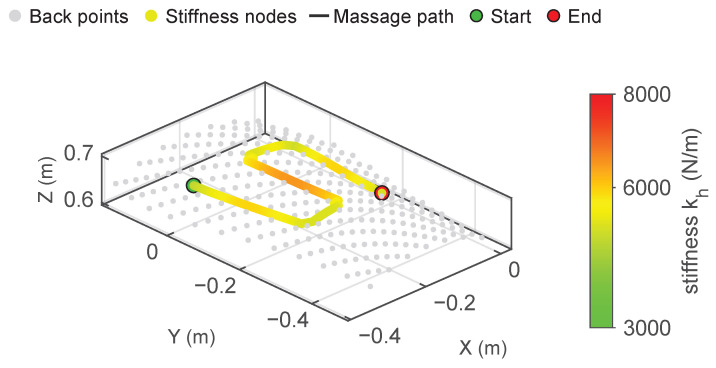
Desired massage trajectory on the simulated human back surface. The gray points denote the discretized back surface, the black curve denotes the planned tangential massage path, and the colored trajectory nodes indicate the local stiffness values along the path. The green and red markers represent the starting and ending points, respectively.

**Figure 4:**
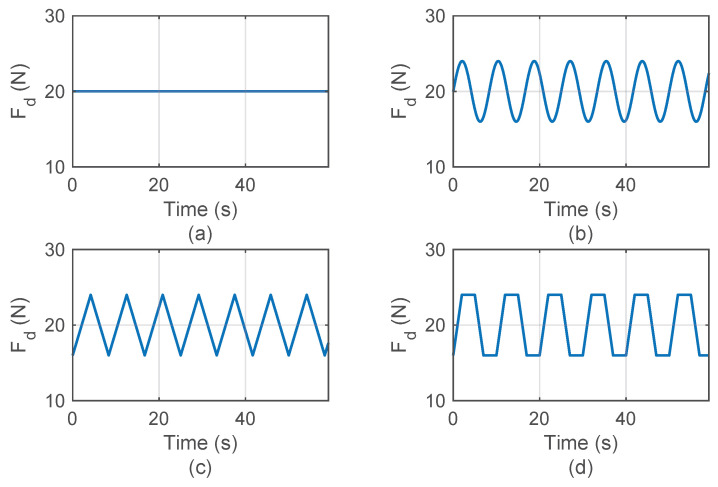
Desired normal-force profiles for four representative TCM massage manipulations: (**a**) pressing/An with a constant force profile, (**b**) kneading/Rou with a sinusoidal force profile, (**c**) rubbing/Mo with a triangular force profile, and (**d**) percussion/Kou with a trapezoidal force profile.

First, the desired trajectory profiles of TCM massage manipulations are given in Figure 3. Meanwhile, four representative Traditional Chinese Medicine (TCM) massage manipulations were mapped into desired normal-force trajectories, as shown in Figure 4. The pressing manipulation, denoted as An, is represented by a constant desired force of 20 N, which mainly evaluates steady-state force regulation and residual force error elimination. The kneading manipulation, denoted as Rou, is represented by a sinusoidal desired force varying around 20 N, which evaluates the tracking bandwidth and phase consistency of the controller. The rubbing manipulation, denoted as Mo, is represented by a triangular force profile, where the desired force contains slope discontinuities at the turning points. This case is used to test whether the controller can suppress force oscillations induced by non-smooth force references. Finally, the percussion manipulation, denoted as Kou, is represented by a trapezoidal force profile with rapid transitions between low-force and high-force segments, which evaluates transient impact dissipation and overshoot suppression.

The mapping between the massage manipulation, desired force profile, massage motion, and corresponding control challenge is summarized in Table 1. Compared with the preliminary simulation design, this setting is more consistent with the physical characteristics of massage tasks because each manipulation contains both a specific force waveform and a distinct motion pattern. In the simulation, the end-effector was commanded to move along a pre-defined massage path on the back surface. The normal force correction generated by the admittance model was superimposed onto the nominal massage trajectory through the force–position decoupling matrix in (4), so that tangential motion tracking and normal force regulation could be evaluated simultaneously.

**Figure 5:**
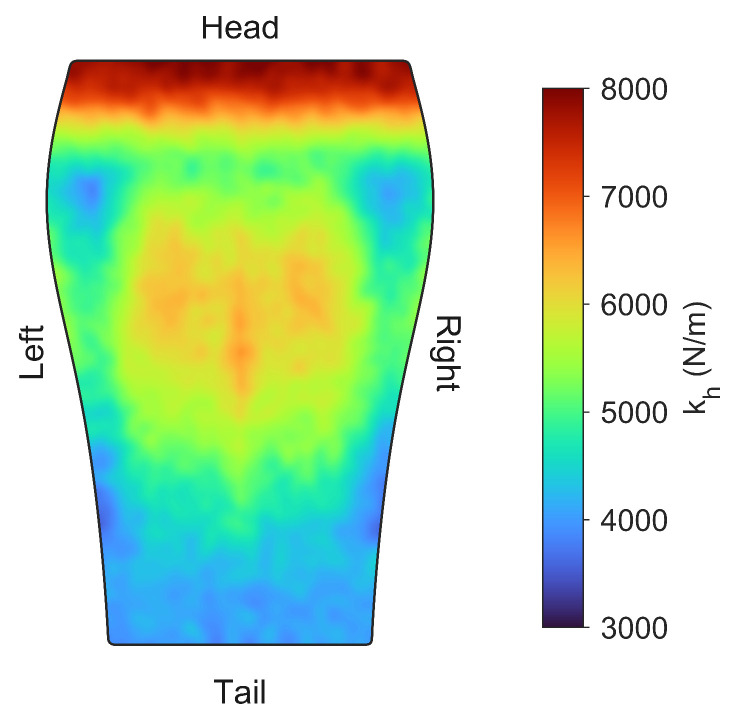
Spatial stiffness distribution of the simulated human back surface. The color map represents the local normal stiffness of different back regions, covering both low-stiffness soft-tissue-like areas and high-stiffness regions near the spine and scapula. This stiffness distribution is used only to construct the simulation environment and is not provided to the controller.

Second, to emulate the heterogeneous mechanical characteristics of the human back, the contact environment was modeled as a curved surface with spatially varying local effective contact stiffness. As shown in Figure 5, the gray point cloud represents the discretized back surface, while the massage trajectory nodes are colored according to the local stiffness. The stiffness distribution covers low-stiffness soft-tissue regions and high-stiffness regions that approximate areas close to the scapula and spine. Based on reported in vivo lumbar stiffness measurements, the stiffness distribution used in the main simulations was set within the range $3.0\times 10^{3}$ N/m to $8.0\times 10^{3}$ N/m. This range corresponds to approximately 3.0–8.0N/mm, which is consistent with reported lumbar posterior-to-anterior stiffness values measured in asymptomatic subjects [51] and external lumbar bulk stiffness measurements obtained through instrumented indentation testing [52].

The stiffness values were unknown to the controller and were used only to construct the simulated interaction environment. Therefore, the controller had to regulate the contact force only through force-sensor feedback, without prior knowledge of the environment stiffness.

For comparison, a fixed-parameter admittance controller was used as the baseline. The baseline controller adopted the same stiffness-free admittance structure as the proposed method, but its damping coefficient was fixed during the whole task. Its normal-direction dynamics can be written as

(30) $$m_{d}{\ddot{z}}_{c}\left(t\right)+b_{\mathrm{fix}}{\dot{z}}_{c}\left(t\right)=e_{f}\left(t\right),$$

where $b_{\mathrm{fix}}$ was tuned as a compromise between response speed and contact stability. In contrast, the proposed method (19) updated the damping coefficient online according to the force tracking error and its derivative.

The main simulation and controller parameters were set as follows. The virtual mass was selected as $m_{d}=1$, the fixed damping coefficient of the baseline admittance controller was set to $b_{\mathrm{fix}}=1000$, and the minimum damping coefficient of the proposed adaptive controller was set to $b_{\mathrm{base}}=1000$. The adaptive damping gains were chosen as α=1.5 and β=2. The control sampling period was Δt=0.001s, corresponding to a control frequency of 1kHz. To emulate force-sensor measurement uncertainty, bounded force noise within ±0.8N was added to the measured contact force. The spatial stiffness of the simulated human back surface varied within a range of approximately $5.0\times 10^{3}$–$1.4\times 10^{4}\;N/m$, covering both soft-tissue-like and high-stiffness contact regions.

For a fair comparison, the virtual mass $m_{d}$, sampling period, desired force trajectory, massage path, robot model, force sensor model, and human back stiffness distribution were kept identical for both controllers. The difference between the two groups lies only in whether the damping parameter was fixed or adaptively regulated.

The force tracking performance was evaluated using four indices. The root mean square error (RMSE), mean absolute error (MAE), maximum absolute error, and the squared correlation coefficient $r^{2}$ are defined as

(31) $$\mathrm{RMSE}=\sqrt{\frac{1}{N}\sum_{i=1}^{N}{\left(F_{\mathrm{ext},i}-F_{d,i}\right)}^{2}},$$

(32) $$\mathrm{MAE}=\frac{1}{N}\sum_{i=1}^{N}\left|F_{\mathrm{ext},i}-F_{d,i}\right|,$$

(33) $$e_{\max}=\max_{1\leq i\leq N}\left|F_{\mathrm{ext},i}-F_{d,i}\right|,$$

and

(34) $$r^{2}={\left(\frac{\sum_{i=1}^{N}\left(F_{d,i}-{\bar{F}}_{d}\right)\left(F_{\mathrm{ext},i}-{\bar{F}}_{\mathrm{ext}}\right)}{\sqrt{\sum_{i=1}^{N}{\left(F_{d,i}-{\bar{F}}_{d}\right)}^{2}}\sqrt{\sum_{i=1}^{N}{\left(F_{\mathrm{ext},i}-{\bar{F}}_{\mathrm{ext}}\right)}^{2}}}\right)}^{2}.$$

For the pressing task, $F_{d}$ is a constant signal; therefore, the variance of the desired force is zero and $r^{2}$ is not reported for this case.

### 5.3. Simulation Results

**Figure 6:**
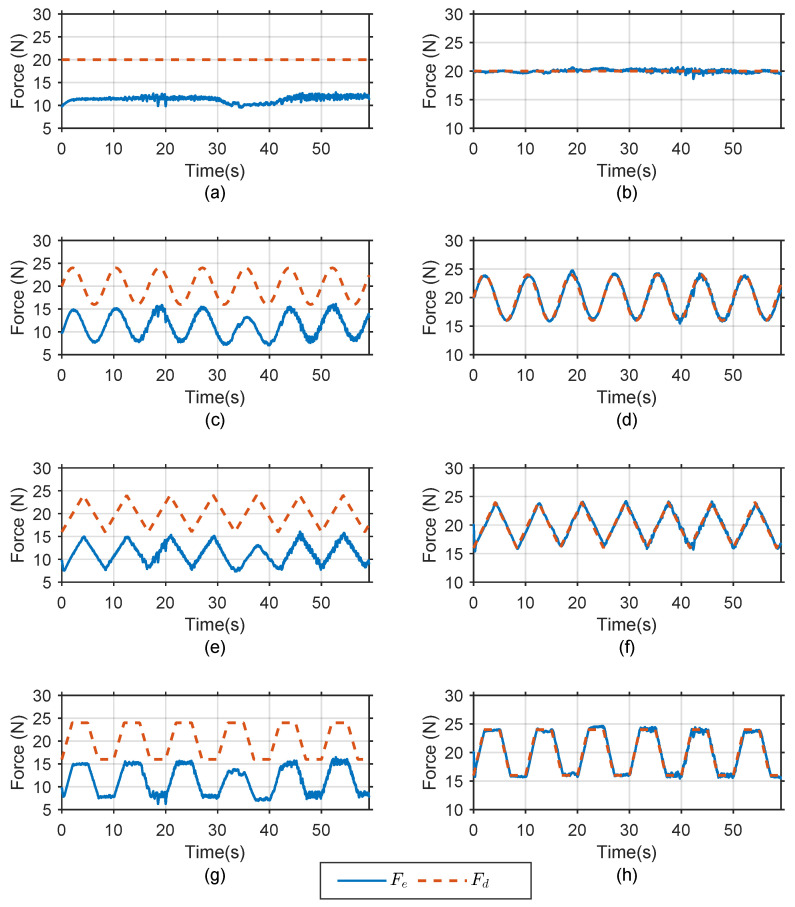
Force tracking comparison between the fixed-parameter admittance controller and the proposed adaptive variable damping admittance controller. The left column (**a**,**c**,**e**,**g**) shows the fixed-parameter method, and the right column (**b**,**d**,**f**,**h**) shows the proposed method. The rows correspond to constant, trapezoidal, triangular, and sinusoidal desired force profiles, respectively.

Figure 6 compares the force tracking responses of the fixed-parameter admittance controller and the proposed adaptive variable damping admittance controller. The left column shows the results of the fixed-parameter controller, while the right column shows the results of the proposed controller under the same desired force trajectories and the same massage path. This comparison clearly demonstrates that the fixed-parameter controller suffers from an inherent transparency–stability trade-off. When the damping is selected to maintain stability during contact with high-stiffness regions, the force response becomes sluggish and the measured force cannot follow the desired force amplitude accurately. This phenomenon is especially obvious in the trapezoidal, triangular, and sinusoidal force tracking cases, where the actual force generated by the fixed-parameter controller presents considerable amplitude attenuation and phase lag.

For the pressing task, the fixed-parameter controller fails to reach the desired 20 N force level and exhibits a clear steady-state deviation. In contrast, the proposed method rapidly converges to the desired force and maintains a measured force close to the reference during the whole massage process. This verifies that the stiffness-free admittance model can remove the steady-state force offset, while the adaptive damping law prevents excessive oscillation during contact with spatially varying stiffness.

For the trapezoidal and triangular force profiles, the superiority of the proposed method becomes more evident. The fixed-parameter method produces a delayed and attenuated force response, especially near the rising and falling edges of the desired force. This indicates that a single damping value cannot simultaneously guarantee fast force tracking and stable contact. By contrast, the proposed controller increases the damping when the force error and its derivative indicate an expanding error trend, thereby dissipating transient contact energy. When the force error begins to converge, the adaptive damping is automatically reduced, which releases the response bandwidth and allows the measured force to follow the desired profile more closely. As a result, the proposed method maintains high tracking fidelity at both the plateau segments and the transition segments.

For the sinusoidal force profile, the fixed-parameter controller shows obvious amplitude loss and tracking lag, whereas the proposed method keeps the actual force nearly synchronized with the desired force. This result confirms that the adaptive variable damping strategy does not merely suppress impact peaks; it also improves the dynamic tracking capability for continuously varying massage forces. Therefore, the proposed method is suitable for both quasi-static massage actions and dynamic rhythmic manipulations.

**Figure 7:**
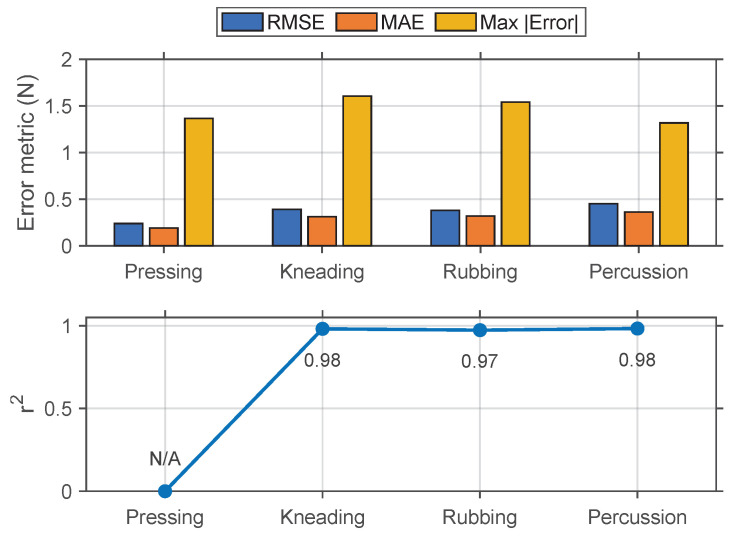
Quantitation of absolute force error of the proposed method: (**Top**) RMSE, MAE, and maximum absolute force error; (**Bottom**) squared correlation coefficient $r^{2}$ between the desired and actual force trajectories.

The quantitative force tracking performance of the proposed method is summarized in Figure 7. Across the four massage modalities, the RMSE remains below 0.42 N and the MAE remains below 0.32 N. The maximum absolute error is lower than 1.8 N for all tasks, which indicates that the proposed method can effectively suppress large transient force deviations even when the desired force contains sharp transitions. Moreover, the $r^{2}$ values for the three non-constant desired force profiles are higher than 0.97, demonstrating strong correlation between the desired and measured force trajectories.

Overall, these results verify the effectiveness of the proposed adaptive variable damping admittance controller in three aspects. First, the controller achieves accurate force tracking under four different desired force profiles, rather than being limited to a constant-force task. Second, it preserves stable contact when the massage head moves along a curved back surface with spatially varying stiffness. Third, compared with the fixed-parameter admittance controller, the proposed method substantially reduces force attenuation, steady-state deviation, and transient tracking lag. These findings demonstrate that the proposed controller is better suited for robotic back massage, where the robot must simultaneously handle non-uniform human tissue stiffness, continuous tangential motion, and time-varying therapeutic force commands.

Table 2 presents the comparison of the relative force tracking error between the conventional admittance controller and the proposed adaptive damping admittance controller under four representative massage techniques. The relative error is defined as

(35) $$e_{r}=100\%\times \frac{F_{\mathrm{ext}}-F_{d}}{F_{d}},$$

where $F_{\mathrm{ext}}$ denotes the actual interaction force and $F_{d}$ denotes the desired force. As shown in Table 2, the proposed controller significantly reduces the force tracking error for all massage techniques. Specifically, the relative error decreases from 52.472%, 56.261%, 57.327%, and 60.987% to 8.5832%, 7.1429%, 8.8851%, and 10.7810% for the pressing, kneading, rubbing, and percussion massage techniques, respectively. Correspondingly, the errors are reduced by 87.642%, 87.304%, 84.501%, and 82.323%.

These results demonstrate that the proposed adaptive damping admittance controller achieves substantially improved force tracking performance and exhibits strong adaptability across different massage motions. The consistently large reduction in relative error indicates that the proposed method can effectively compensate for variations in contact conditions and maintain more accurate human–robot interaction forces than the conventional admittance controller.

### 5.4. Stiffness Sensitivity and Robustness Analysis

To investigate the influence of environmental stiffness on the force-control performance and to evaluate the robustness of the proposed controller, simulations are conducted under four representative contact stiffness conditions: $k_{h}$ = {3000, 5000, 8000, 14,000} N/m. The first three stiffness values correspond to the clinically representative range adopted in the main simulation, while the highest value (14,000 N/m) is introduced as a high-resistance contact condition to emulate localized interactions near bony anatomical structures, regions of elevated muscle tone, or abrupt stiffness variations.

**Figure 8:**
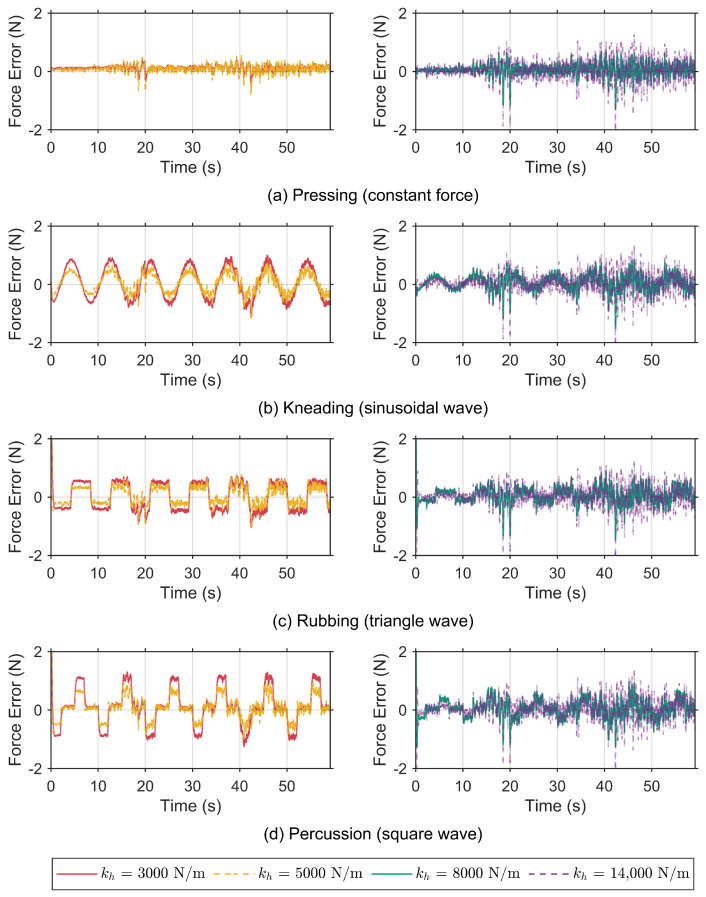
Comparison of force tracking error under various stiffness conditions.

For each stiffness condition, a constant desired contact force was applied, and the actual interaction force generated by the controller was recorded. Figure 8 compares the desired force and the measured force under different stiffness conditions. The results show that the proposed adaptive damping admittance controller achieves stable force tracking over the entire stiffness range. Although the transient response exhibits slight variations as the environmental stiffness increases, the actual force remains close to the desired force in all cases, indicating satisfactory adaptability to different contact environments.

**Figure 9:**
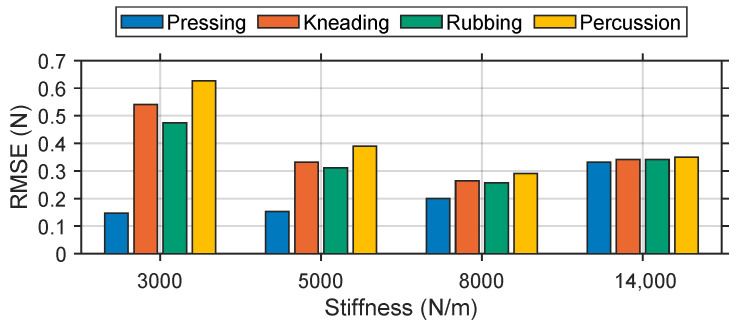
RMSE of absolute force error under various stiffness conditions.

To quantitatively evaluate the tracking performance, the root-mean-square error (RMSE) of the force tracking error was calculated for each stiffness condition. The comparison results are illustrated in Figure 9. It can be observed that the RMSE values remain within a narrow range despite a more than fourfold increase in environmental stiffness from 3000N/m to 14,000 N/m. This result demonstrates that the proposed controller is not strongly dependent on a specific stiffness assumption and maintains consistent force-regulation performance under both clinically representative and high-resistance contact conditions.

Therefore, the proposed adaptive damping admittance control strategy exhibits good robustness against variations in environmental stiffness and is capable of maintaining stable human–robot interaction across a broad range of contact scenarios.

### 5.5. Discussion

Synthesizing the in-depth analysis of the four typical massage modalities mentioned above, the control system proposed in this paper profoundly addresses a series of engineering pain points.

Firstly, addressing the constraints of closed position control in industrial collaborative robots, this study proved that by discarding the virtual stiffness term and nesting an implicit Euler discretized admittance model, it is possible to completely eradicate steady-state force tracking errors caused by environmental position drift without altering the underlying servo loops. This design entirely converts force control into kinematic corrections on the outer loop, holding immense engineering utility value.

Secondly, the adaptive variable damping strategy based on force error and its rate of change perfectly reconciles the “Transparency–Stability Trade-off” in pHRI [53]. This strategy not only rigorously guarantees the global asymptotic stability of the closed-loop system mathematically via Lyapunov theory, but its physical behavior also mimics a biological neural reflex mechanism in humans. That is, a masseur instinctively tenses their arm muscles to increase impedance and dissipate impact when encountering hard bones, while relaxing their arm to sensitively follow undulations when moving over soft muscle tissues [54]. The adaptive penalty term $\beta \max(0,e_{f}{\dot{e}}_{f})$ presented in (12) perfectly replicates this physiological mechanism in digital space.

Finally, relying on the high-quality geometric posture interpolations provided by dual quaternions, this force control law effectively decouples physical interference between tangential macro-movements and normal micro-force control. It endows the robotic arm with intrinsic safety when executing high-frequency, large-load, time-varying massage tasks on complex unstructured curved surfaces, laying a solid validation foundation for clinical medical applications.

## 6. Conclusions

In summary, addressing the demands for compliant interaction and dynamic force tracking in multi-mode TCM massage tasks performed by medical rehabilitation robots, this paper proposes an adaptive variable damping admittance control system designed for robotic arms with closed position-control architectures. Targeting the stiffness uncertainties and time-varying morphology brought about by the unstructured environment of the human back, this research first constructs a stiffness-free second-order admittance model in Cartesian space, and applies implicit Euler discretization, reducing steady-state force tracking errors and improving numerical robustness under high-stiffness contact. Furthermore, a nonlinear adaptive damping regulation law driven by real-time force tracking errors and their derivatives is designed. Using Lyapunov theory, the closed-loop stability and force-error convergence properties of the proposed controller are analyzed under positive bounded environment stiffness.

A joint simulation platform based on MATLAB and CoppeliaSim using the ZeroMQ protocol was established to evaluate the practical performance of the proposed algorithm. Traditional massage techniques, including “pressing, kneading, rubbing, and percussion”, were deconstructed into diverse time-varying target force signals such as constant, sinusoidal, triangular, and trapezoidal waveforms. The simulation results demonstrate that the proposed algorithm improves trajectory geometric compliance and transient force response across planar, inclined, and complex bionic 3D curved surfaces compared with traditional fixed-parameter admittance control. With reduced overshoot and attenuated force oscillations, the system can better reproduce the mechanical characteristics of multi-mode TCM massage and improve the force tracking robustness of robotic massage under unknown and spatially varying stiffness conditions.

Looking ahead to future work, we intend to advance from virtual validation to physical prototype experiments. On the one hand, the proposed control algorithm will be implemented on a real Franka robotic arm to further evaluate and compensate for the effects of physical joint flexibility, friction nonlinearity, actuator saturation, communication delay, and high-frequency sensor noise on force control performance. On the other hand, to adapt to more realistic and dynamic interaction environments, RGB-D visual sensing and multi-modal data fusion will be considered to construct feedforward predictive compensation mechanisms for patient respiratory rhythms and autonomous withdrawal behaviors, thereby promoting the practical implementation of intelligent multi-mode TCM massage robotic systems.

## Figures and Tables

**Table 1:** Mapping of TCM massage manipulations to simulation tasks.

TCM Modality	Massage Motion	Desired Force	Main Control Challenge
Pressing/An	Local pressing or slow normal loading	Constant force	Steady-state force accuracy and elimination of residual force offset.
Kneading/Rou	Smooth cyclic manipulation along the back surface	Sinusoidal force	Tracking bandwidth, phase consistency, and suppression of periodic force lag.
Rubbing/Mo	Continuous tangential rubbing along the massage path	Triangular force	Handling non-smooth reference slopes and reducing oscillations near turning points.
Percussion/Kou	Repeated impact-like normal loading	Trapezoidal force	Transient impact dissipation, overshoot suppression, and fast recovery after force jumps.

**Table 2:** Force tracking RMSE comparison under different massage techniques.

Method	Pressing	Kneading	Rubbing	Percussion
Conventional Method	52.472%	56.261%	57.327%	60.987%
Proposed Method	8.5832%	7.1429%	8.8851%	10.7810%
Improvement	87.642%	87.304%	84.501%	82.323%

## Data Availability

Data are available upon request.

## Abbreviations

The following abbreviations are used in this manuscript:

AVIC	Adaptive Variable Impedance Control
DLS	Damped Least Squares
DOF	Degree of Freedom
DRL	Deep Reinforcement Learning
HRV	Heart Rate Variability
LBP	Low Back Pain
MAE	Mean Absolute Error
ODI	Oswestry Disability Index
pHRI	Physical Human–Robot Interaction
PID	Proportional–Integral–Derivative
PNS	Parasympathetic Nervous System
RCTs	Randomized Controlled Trials
RL	Reinforcement Learning
RMSE	Root Mean Square Error
ROM	Range of Motion
TCM	Traditional Chinese Medicine
TLF	Thoracolumbar Fascia
URDF	Unified Robot Description Format
VAC	Variable Admittance Control
VAS	Visual Analog Scale
VIC	Variable Impedance Control
